# Rk1, a Ginsenoside, Is a New Blocker of Vascular Leakage Acting through Actin Structure Remodeling

**DOI:** 10.1371/journal.pone.0068659

**Published:** 2013-07-22

**Authors:** Yong-Sun Maeng, Sony Maharjan, Jeong-Hun Kim, Jeong-Hill Park, Young Suk Yu, Young-Myoung Kim, Young-Guen Kwon

**Affiliations:** 1 Department of Biochemistry College of Life Science and Biotechnology, Yonsei University, Seoul, Republic of Korea; 2 Corneal Dystrophy Research Institute and Department of Ophthalmology, Yonsei University College of Medicine, Seoul, Republic of Korea; 3 Department of Ophthalmology, College of Medicine, Seoul National University, Seoul Artificial Eye Center, Clinical Research Institute, Seoul National University Hospital, Seoul, Republic of Korea; 4 Research Institute of Pharmaceutical Science, College of Pharmacy, Seoul National University, Seoul, Republic of Korea; 5 Vascular System Research Center, Kangwon National University, Kangwon-Do, Republic of Korea; University of Southampton, United Kingdom

## Abstract

Endothelial barrier integrity is essential for vascular homeostasis and increased vascular permeability and has been implicated in many pathological processes, including diabetic retinopathy. Here, we investigated the effect of Rk1, a ginsenoside extracted from sun ginseng, on regulation of endothelial barrier function. In human retinal endothelial cells, Rk1 strongly inhibited permeability induced by VEGF, advanced glycation end-product, thrombin, or histamine. Furthermore, Rk1 significantly reduced the vessel leakiness of retina in a diabetic mouse model. This anti-permeability activity of Rk1 is correlated with enhanced stability and positioning of tight junction proteins at the boundary between cells. Signaling experiments revealed that Rk1 induces phosphorylation of myosin light chain and cortactin, which are critical regulators for the formation of the cortical actin ring structure and endothelial barrier. These findings raise the possibility that ginsenoside Rk1 could be exploited as a novel prototype compound for the prevention of human diseases that are characterized by vascular leakage.

## Introduction

The disruption of endothelial barrier integrity leading to increased vasopermeability contributes to many pathological processes, including various inflammatory diseases, acute lung injury, and diabetic retinopathy [1,2]. Endothelial permeability is tightly controlled by cell-cell junctions, including adherent junctions (AJs) and tight junctions (TJs), between neighboring endothelial cells [3,4]. TJs consist of a number of proteins, including occludin, claudins, junctional adhesion molecules (JAMs), and zonula occludins (ZO). Occludin, claudins, and JAMs are major integral transmembrane proteins with adhesive properties, and are believed to be responsible for the formation of a tight seal between two apposing endothelial membranes of adjacent cells [3]. Occludin and claudins form homodimeric bridges, and ZOs connect these integral transmembrane proteins to actin filaments [5–7]. Dynamic regulation of perijunctional actin has been suggested to control paracellular permeability by affecting the stability of TJs closely connected to the actin cytoskeleton, either directly or indirectly [8,9]. In fact, there is ample ultrastructural evidence to implicate the temporal expression, dynamic organization, and spatial distribution of the actin cytoskeleton in the alteration of TJ complexes under various conditions [10]. Therefore, actin is likely to play a critical role in modulating the integrity of TJs, and thus, endothelial permeability.

The reorganization of the actin cytoskeleton into the cortical actin ring and the concomitant redistribution of TJ proteins to the cell periphery is an essential event in endothelial barrier enhancement. Several molecules have been suggested to be important for the formation of the cortical actin ring [11]. Phosphorylated myosin light chain (p-MLC), as well as its kinase, myosin light chain kinase (MLCK), were observed to be distributed in the cortical region during endothelial cell (EC) barrier enhancement induced by sphingosine-1-phosphate (S1P) [12,13], suggesting a potential role for spatially defined MLCK activation in regulating endothelial barrier function. MLC phosphorylation at the cortical region may promote the interaction of actin filaments and myosin, stabilizing the cortical actin ring structures, and thereby increasing the stability of TJ protein complexes in the cell periphery [11]. Cortactin, an F-actin binding protein, has also been implicated in cortical actin rearrangement [14]. Cortactin translocates from the cytoplasm to the periphery in response to many of the same stimuli that induce its tyrosine phosphorylation, including growth factor treatment, integrin binding and bacterial entry. These events also lead to activation of Rac, which are responsible for controlling the formation of cortical actin networks. Rac activation is required for cortactin translocation to the cell periphery, and cortactin disperses from cortical actin networks following treatment with the F-actin disrupting drugs. Therefore, Cortactin translocation to the cortical actin is closely associated with Rac-mediated actin assembly and enhanced endothelial barrier function [13].

Diabetic retinopathy (DR) is one of the most common vascular retinopathies and a leading cause of legal blindness in working-age adults [15]. The earliest sign of DR is leakage from retinal vessels due to breakdown of the blood-retinal barrier (BRB), which is followed by retinal edema and finally endothelial cell proliferation [16]. The BRB is a selective endothelial barrier of well-differentiated microvessels of the eye. The disruption of the BRB occurs during the earliest period of vascular retinopathy, which can be recovered before the irreversible effect of angiogenesis characteristic of proliferative vascular retinopathy [17]. VEGF is known to play an important role in BRB breakdown by altering tight junction integrity and the cytoskeleton organization of endothelial cells, leading to increased permeability during the pathogenesis of DR [18,19]. Therapies targeting this early and reversible stage of BRB breakdown remain to be developed.

Ginseng generally refers to the medicinal herb derived from the roots of species of the genus *Panax*. Ginseng, long used in traditional oriental medicine, is known to have a wide range of medicinal effects, including tonic, immunomodulatory, anti-mutagenic, adaptogenic, and anti-aging activities [20,21]. In addition, several clinical studies have shown that *Panax* ginseng improves psychological physiological function and conditions associated with diabetes [20]. The main active components responsible for the medicinal effects of *Panax* ginseng are ginsenosides, which are triterpene saponins. The ginsenoside composition of ginseng can vary depending on how it is processed. Sun ginseng, recently developed by heat-treatment at a temperature and pressure higher than those applied to the conventional preparation of red ginseng, was shown to contain ginsenosides enriched with Rg3, Rg5, Rk1, Rg6, Rh2, Rh3, Rh4, and Rs3, which are either entirely absent or present only in trace amounts in conventional white or red ginseng [22,23].

In this study, we investigated the potential pharmacological activities of ginsenosides in preventing the pathological loss of vascular integrity. Surprisingly, our data show that Rk1, a derivative of natural ginsenosides, has a strong anti-vascular permeability activity against major pathological vascular permeability factors, such as VEGF and Advanced Glycation End-product (AGE). Moreover, the activity of Rk1 is correlated with its prominent effects on the robust formation of the cortical actin ring, which is coordinated by MLC and cortactin, and the stabilization of TJ complexes at cell junctions.

## Materials and Methods

### Cell culture

Primary human retina microvascular endothelial cells (HRECs) were purchased from the Applied Cell Biology Research Institute and were initiated by elutriation of dispase dissociated normal human retina tissue. These cells grown on attachment factor-coated plates in complete medium (Cell Systems, Kirkland, WA) or in M199 medium supplemented with 20% FBS, 3 ng/ml basic FGF (Millipore, Bedford, MA), and 10 U/ml heparin (Sigma, St. Louis, MO).

### Source of ginsenosides

Ginsenosides Rb1, Rg1, Rk1 were isolated as previously described [22]. Briefly, raw and steamed ginsengs were refluxed with methanol for 6 h. After removal of methanol with an evaporator, the aqueous residue was extracted with butanol. Then, the butanol fraction was subjected to silica gel chromatography or prep-HPLC for the isolation of ginsenoside. Chemical structure of isolated ginsenoside was confirmed by NMR and MS. For the qualitative and quantitative analysis of ginsenosides, an HPLC-evaporative light scattering detector (ELSD) was used. The purity of ginsenosides was decided by calculation of the peak area from HPLC-ELSD data. The purity of the ginsenosides and percentage of various ginsenosides in mixtures used in our experiments were Rb1 (>85%), Rg1 (97%), and Rk1 (>85%). Ginsenosides were dissolved in dimethylsulfoxide (DMSO).

### [^3^H] sucrose permeability assay

HRECs were plated onto a Transwell filter (Corning Costar, Acton, MA). After reaching confluence, HRECs were incubated with M199 containing 1% FBS for 3 h and pretreated for 40 min with or without Rb1, Rg1 or Rk1 (10 µg/ml) prior to stimulation with VEGF (20 ng/ml) (Upstate Biotechnology, Lake Placid, NY), Thrombin (10 units/ml) or Histamine (100 µM) (Sigma-Aldrich, St Louis, MO) for 1 h or 5 µM AGE-BSA for 10 h. HRECs were pretreated for 30 min with or without 1C11 (10 µg/ml) prior to stimulation with 5 µM AGE-BSA for 10 h. 50 µl (0.8 µCi [0.0296 MBq]/ml) of [^3^H] sucrose (1 µCi [0.037 MBq]/ μl; Amersham Pharmacia) were added to the upper compartment. The amount of radioactivity that diffused into the lower compartment was determined after 30 min with a liquid scintillation counter (PerkinElmer, Wallac, Finland). Each experimental point was performed at least four times.

### Triton X-100 extraction of TJ proteins

HRECs plated in 6-well plates were serum starved in M199 containing 1% FBS for 6 h. They were then pretreated for 40 min with or without Rk1 (10µg/ml) prior to stimulation with VEGF (20 ng/ml) for 1 h or pretreated for 30 min with or without 1C11 (10 µg/ml) prior to stimulation with 5 µM AGE-BSA for 10 h, and fractionated in cytoskeleton-stabilizing buffer (10 mM HEPES [pH 7.4], 250 mM sucrose, 150 mM KCl, 1 mM EGTA, 3 mM MgCl_2_, 1 x protease inhibitor cocktail [Roche Diagnostics Modular, Germany], 1 mM Na3VO4, 0.5% Triton X-100) by centrifugation at 15,000 *g* for 15 min. The proteins in the Triton X-100-insoluble and soluble fractions were analyzed by Western blotting.

### Immunofluorescence staining and immunohistochemistry

Confluent HRECs were pretreated for 40 min with or without Rk1 (10 µg/ml) prior to stimulation with 20 ng/ml VEGF (1 h) or 5 µM AGE-BSA (10 h). Confluent HRECs were treated with Rk1 (10 µg/ml) for 30min or 1h. HRECs were fixed in 4% paraformaldehyde for 20 min and permeabilized in 0.1% Triton X-100/PBS, then pre-incubated with a blocking solution of PBS containing 5% normal goat serum and 0.05% Tween-20. Cells were then incubated for 2 h in primary antibody [mouse anti-ZO-1, anti-ZO-2, anti-occludin (Zymed Laboratories Inc., CA), anti-cortactin (Upstate Biotechnology, Lake Placid, NY), and anti-pMLC2 (Cell signaling, Beverly, MA)]. Reactions were visualized by fluorescein-conjugated anti-mouse (Vector Lab, CA).

Confluent HRECs were pretreated for 40 min with or without Rk1 (10 µg/ml) prior to stimulation with 20 ng/ml VEGF (1 h) or 5 µM AGE-BSA (10 h). Actin filament rearrangement was monitored by Rhodamine Phalloidin staining, which specifically labels F-actin. HRECs monolayers were fixed in 4% paraformaldehyde for 20 min at room temperature, and then washed 3 times with PBS (pH 7.4). Cells were then permeabilized in 0.1% Triton X- 100/PBS and incubated with 0.1 mg/ml of Rhodamine Phalloidin (Molecular Probes, OR) for 1 h. All samples were observed with a fluorescence microscope (Olympus, Olympus IX81-ZDC microscope [Olympus, Tokyo, Japan]).

Diabetic mice were injected with 15 µg Rk1 into the vitreous of one eye and with vehicle in the contra lateral eye. At 24 h after injection, the mouse eyes were then enucleated and immediately fixed in 4% PFA for 4h and washed in PBS. The lens were then removed and the eyes were embedded in paraffin wax for sectioning. Sections of mouse eyes were dewaxed and then rehydrated in Tris-buffered saline (TBS). Sections were then washed in TBS and the antigens unmasked in 0.125% trypsin (10 min at 25 °C), washed again in TBS, and blocked in 5% normal goat serum (20 min) in an antibody dilution buffer consisting of TBS containing 0.01% Triton X-100. Sections were then incubated for overnight in primary antibody [rabbit anti-occludin (Zymed Laboratories Inc., CA)] at 4 °C. Reactions were visualized by FITC-conjugated anti-rabbit (Vector Lab, CA). Nuclei were counterstained with DAPI and are seen in blue. Samples were observed with a fluorescence microscope (Olympus, Olympus IX81-ZDC microscope [Olympus, Tokyo, Japan).

### Perfusion with FITC Dextran

Mice were injected with 50 ng VEGF alone or in combination with 15 µg Rk1 into the vitreous of one eye and with vehicle in the contra lateral eye. At each time point after injection, 150µl FITC-Dextran (30 mg/ml in sterile PBS) was injected into the left ventricle. Mice were deeply anaesthetized using ketamine/xylazine and 40 kDa FITC-Dextran (Sigma-Aldrich, St Louis, MO) (30 mg/ml in sterile PBS) was injected into the left ventricle. The tracer was allowed to circulate for ~2 min and the mouse eyes were then enucleated and immediately fixed in 4% PFA. The mouse eyes were left overnight in PFA. The following day, the mouse retinas were dissected in a Maltese cross-configuration and flat-mounted onto glass slides. The mouse retinas were then viewed by epifluorescence.

### Induction of experimental diabetes

Insulin-deficient diabetes was induced in C57BL/6J mice (8 weeks male) by a single i.p. injection of streptozotocin (STZ; Sigma, St. Louis, MO; 2.5 mg per body-weight (BW(g)), which was dissolved in 0.1 M sodium citrate buffer (pH 4.5) just before injection. Age-matched normal control mice received saline alone. Fasting blood glucose level was evaluated 3 days later. A supplemental STZ injection (1.25 mg per BW(g)) i.p., was given if the fasting blood glucose was below 150 mg dl^−1^. All diabetic (blood glucose level after 8 h fasting >150 mg dl^−1^) and normal mice were maintained with the same diet and water ad libitum for a total of 2 weeks. Each blood sample was taken from the tail vein, and its glucose level was measured with Freestyle Flash Blood Monitoring System (Nipro. Osaka, Japan).

Diabetic mice were injected with Rk1 (1, 5, 10, 15µg) into the vitreous of one eye and with vehicle in the contra lateral eye. At 24 h after injection, 150 µl FITC-Dextran (30 mg/ml in sterile PBS) was injected into the left ventricle. The retinas were then viewed by fluorescence microscopy.

### Semi-quantitative RT-PCR Analysis

HRECs were pretreated for 40 min with or without Rk1 (10 µg/ml) prior to stimulation with VEGF (20 ng/ml) for 6 h or 5 µM AGE-BSA for each time point. Total RNA was obtained from HRECs with a TRIzol reagent kit and 0.5–5 µg RNA was used for RT-PCR. Briefly, target RNA was converted to cDNA by treatment with 200 units of reverse transcriptase and 500 ng of oligo(dT) primer in 50 mM Tris-HCl (pH 8.3), 75 mM KCl, 3 mM MgCl_2_, 10 mM dithiothreitol, and 1 mM dNTPs at 42 °C for 1 h. The reaction was stopped by heating at 70 °C for 15 min. The cDNA mixture (1 µl) was used directly for enzymatic amplification. The polymerase chain reaction was performed in 50 mM KCl, 10 mM Tris-HCl (pH 8.3), 1.5 mM MgCl_2_, 0.2 mM dNTPs, 2.5 units of *Taq* DNA polymerase, and 0.1 µM of primers. Amplification was performed in a DNA thermal cycler (model PTC-200; MJ Research) under the following conditions: denaturation at 94 °C for 5 min for the first cycle and for 30 sec thereafter, annealing at 55 °C for 30 sec, and extension at 72 °C for 30 sec for 28 repetitive cycles. Final extension was at 72 °C for 10 min. All results were normalized to GAPDH mRNA. Primers were as follows: ZO-1 5’-CGACCATTTGAACGCAAGTT-3’ (sense) and 5’-TCAAACTCAGGAGGCTGTGC-3’ (antisense); ZO-2 5’-GAGGAGGTGGGAGAGAGCAG-3’ (sense) and 5’-CTGGCTTGTGCGTTTTGATT-3’ (antisense); Occludin 5’-ATTTATGATGAGCAGCCCCC-3’ (sense) and 5’-TGACCTTCCTGCTCTTCCCT-3’ (antisense); VEGF 5’-GTGGACATCTTCCAGGAGTA-3’ (sense) and 5’-GCGAGTCTGTGTTTTTGC-3’ (antisense); GAPDH 5’-CGCCACAGTTTCCCGGAGGG-3’ (sense) and 5’-CCCTCCAAAATCAAGTGGGG-3’ (antisense). Each experimental condition was performed in quadruplicate.

### Immunoprecipitation

Confluent HRECs were treated with Rk1 (0.1, 1, 5, or 10 µg/ml) for the 30min. Cellular protein from HRECs was incubated with the anti-cortactin antibody (Upstate Biotechnology, Lake Placid, NY) and protein A or G Sepharose in TEG buffer (20 mM Tris-Cl, pH 7.4, 1 mM EDTA, 10% glycerol, and 1 mM dithiothreitol containing 150 mM NaCl and 0.1% Triton X-100) at 4°C for 16 h with constant rotation. Sepharose pellets were collected by centrifugation and washed three times with supplemented TEG buffer. Immunoprecipitates were analyzed by SDS-PAGE, followed by Western blot using the indicated antibodies.

### Animal studies

The mice were housed and maintained under sterile conditions in facilities accredited by the korean Association of Assessment and Accreditation of Laboratory Animal Care. This study was carried out in strict accordance with the recommendations in the Guide for the Care and Use of Laboratory Animals of Yonsei University. The protocol was approved by the Committee on the Ethics of Animal Experiments of the University of Yonsei. All efforts were made to minimize suffering.

### Statistical Analysis

All experiments were repeated at least 3 times. Data are presented as the means ± S.E, and statistical comparisons between groups were performed by one-way ANOVA followed by Tukey’s test.

## Results

### Rk1 inhibits VEGF-induced retinal endothelial permeability

**Figure 1:**
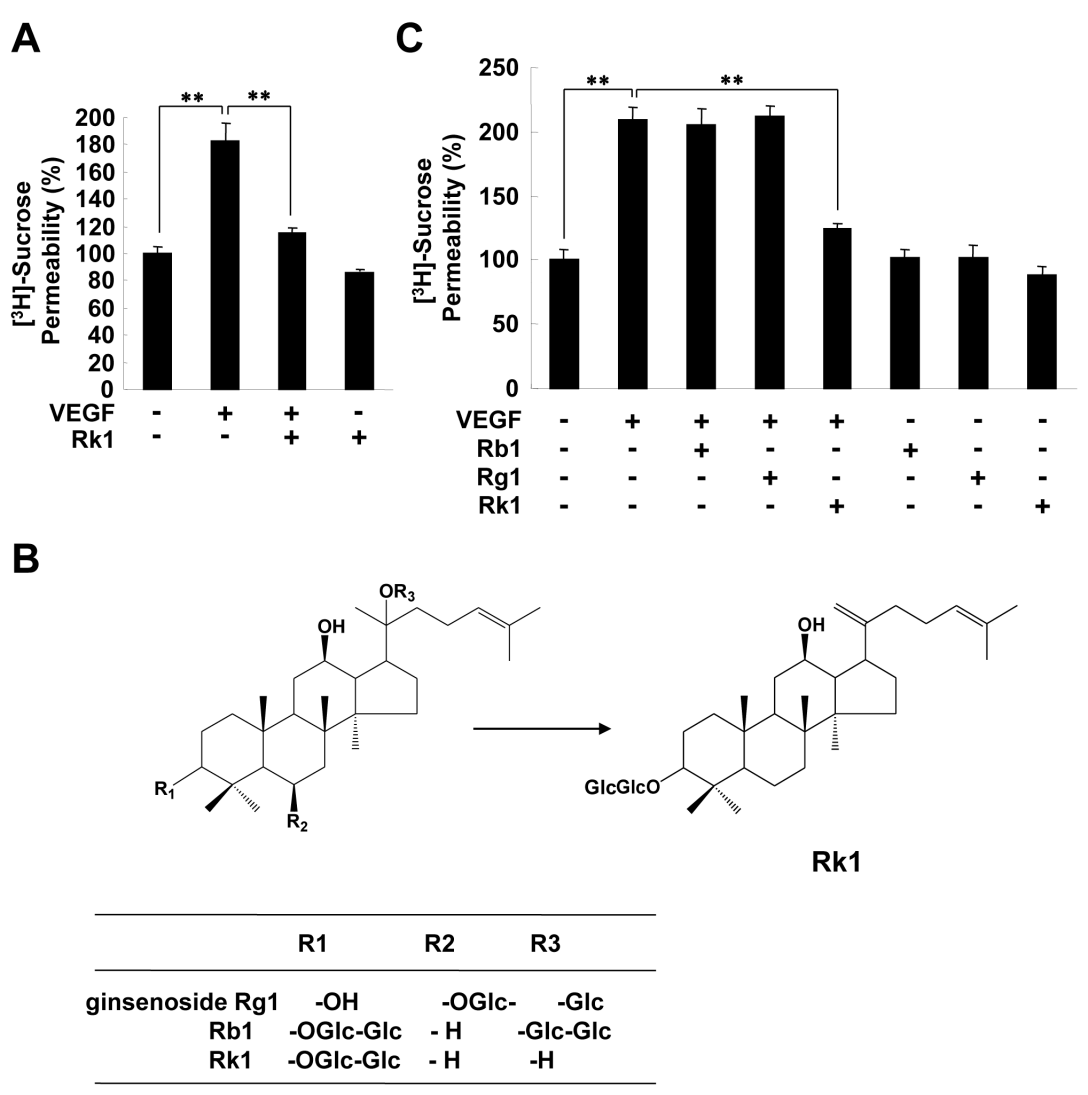
Rk1, but not Rg1 or Rb1, inhibits VEGF-induced retinal endothelial permeability. (**A**) HRECs were plated onto a Transwell filter . After reaching confluence, HRECs were pretreated for 40 min with or without Rk1 (10 µg/ml) prior to stimulation with VEGF (20 ng/ml) for 1 h. HRECs were cultured with 50 µl (0.8 µCi/ml) of [^3^H] sucrose (1 µCi/µl) added to the upper compartment. The amount of radioactivity that diffused into the lower compartment was determined after 30 min with a liquid scintillation counter. (**B**) The structure of gensenosides. (**C**) HRECs were plated onto a Transwell filter. After reaching confluence, HRECs were pretreated for 40 min with or without Rb1, Rg1, and Rk1 (10 µg/ml) prior to stimulation with VEGF (20 ng/ml) for 1 h. HRECs were cultured with 50 µl (0.8 µCi/ml) of [^3^H] sucrose (1 µCi/µl) added to the upper compartment. The amount of radioactivity that diffused into the lower compartment was determined after 30 min with a liquid scintillation counter. All data are presented as the mean ± S.E. from three different experiments performed in duplicate. **, *P* < 0.01 *vs*. VEGF alone.

Vascular integrity is required for functional vasculature maintenance and is lost in many pathological states, including DR. Our preliminary results showed that Rk1, a major component of SG135, which is a mixture of ginsenosides extracted from sun ginseng, has a profound effect on the viability of human retina endothelial cells (HRECs) as compared to other derivatives of ginsenosides. Therefore, we decided to investigate whether Rk1 has a protective effect on the integrity of retina vessels using an *in vitro* HERCs monolayer permeability assay. As shown in Figure 1A, Rk1 was able to inhibit VEGF-induced retinal endothelial permeability. Interestingly, ginsenosides Rb1 and Rg1, which have been reported to have angiogenic-related activities, had no significant effect on VEGF-induced HREC permeability revealing the structural specificity of Rk1 action on endothelial cells (Figure 1B, 1C).

**Figure 2:**
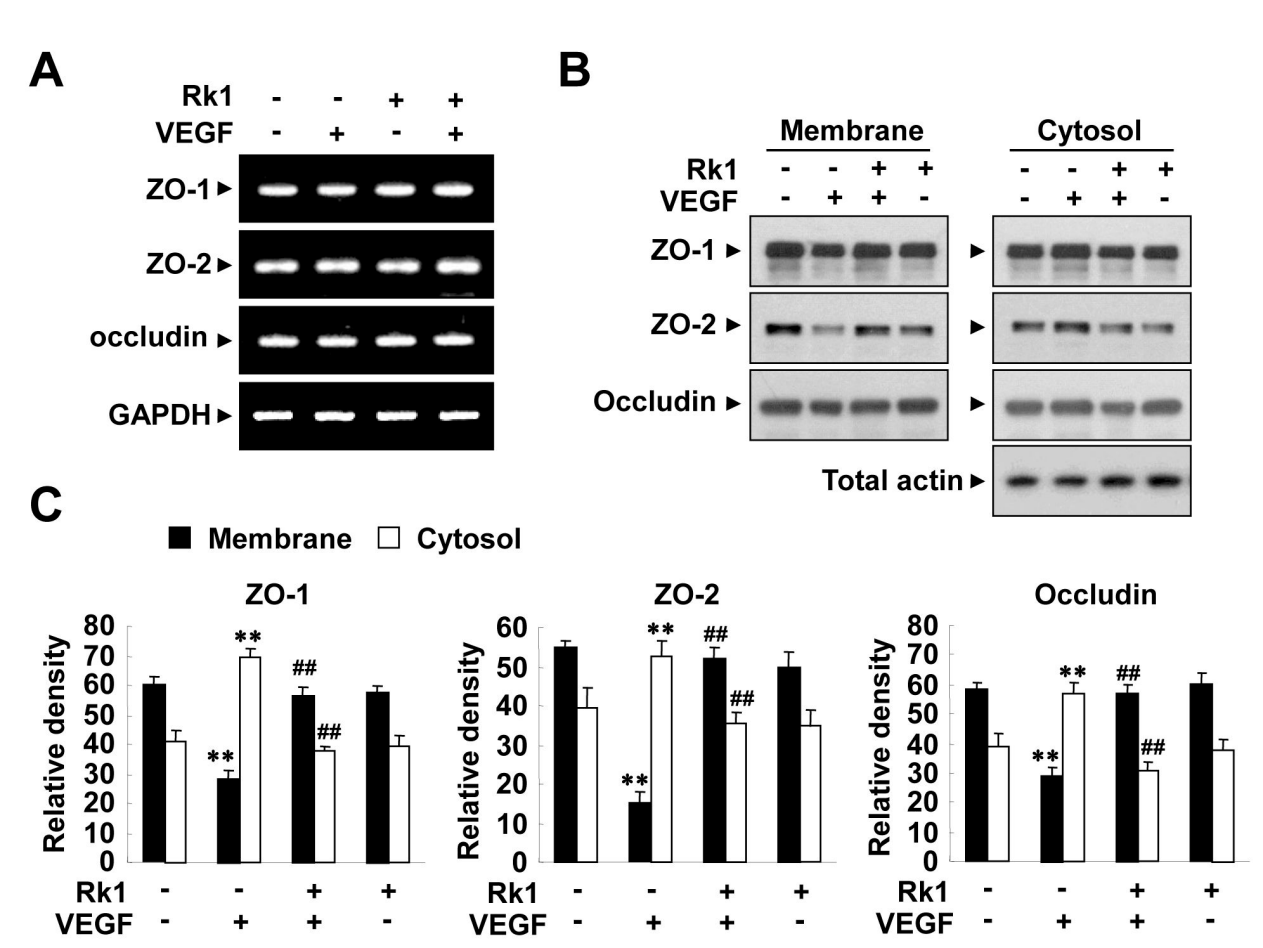
Rk1 inhibits VEGF-induced destabilization of TJ proteins. (**A**) HRECs were pretreated for 40 min with or without Rk1 (10 µg/ml) prior to stimulation with VEGF (20 ng/ml) for 6 h. Total mRNAs were isolated and RT-PCR was performed with specific primers for human ZO-1, ZO-2, and occludin. GAPDH served as an internal control. (**B**) Confluent HRECs were pretreated for 40 min with or without Rk1 (10 µg/ml) prior to stimulation with VEGF (20 ng/ml) for 1 h. Translocation of TJ proteins was assessed as described in “Experimental Procedures”. The Triton X-100-insoluble and soluble fractions were subjected to SDS-polyacrylamide gel electrophoresis followed by Western blot analysis with anti-ZO-1, anti-ZO-2, anti-occludin and anti-Actin. Blots are representative of three independent experiments. (**C**) Densitometric analyses are presented as the relative ratio of tight junction proteins in membrane and cytosol and quantified with NIH ImageJ software. Data are the mean ± S.E. **, *p* < 0.01 *vs*. Control. ^##^, *p* < 0.01 *vs*. VEGF alone.

**Figure 3:**
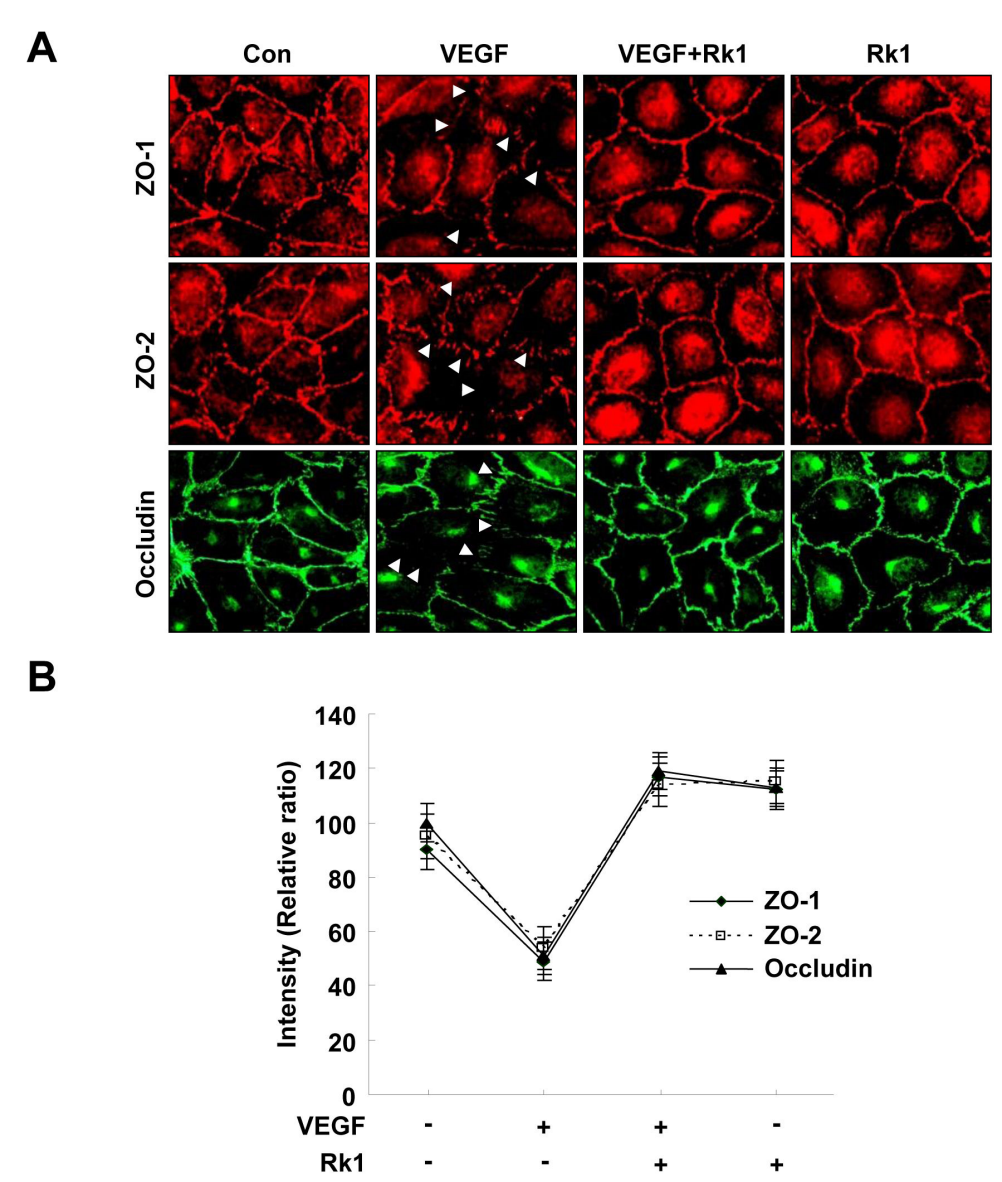
Rk1 effectively inhibits localization of TJ proteins disrupted by VEGF treatment. (**A**) Confluent HRECs were pretreated for 40 min with or without Rk1 (10 µg/ml) prior to stimulation with VEGF (20 ng/ml) for 1 h. The cells were then fixed and stained with anti-ZO-1, anti-ZO-2, and anti-occludin antibodies. Arrowheads indicate disruption of tight junction proteins. (**B**) Tight junction protein intensity at cell borders was analyzed and quantified with NIH ImageJ software. Data are the mean ± S.E.

**Figure 4:**
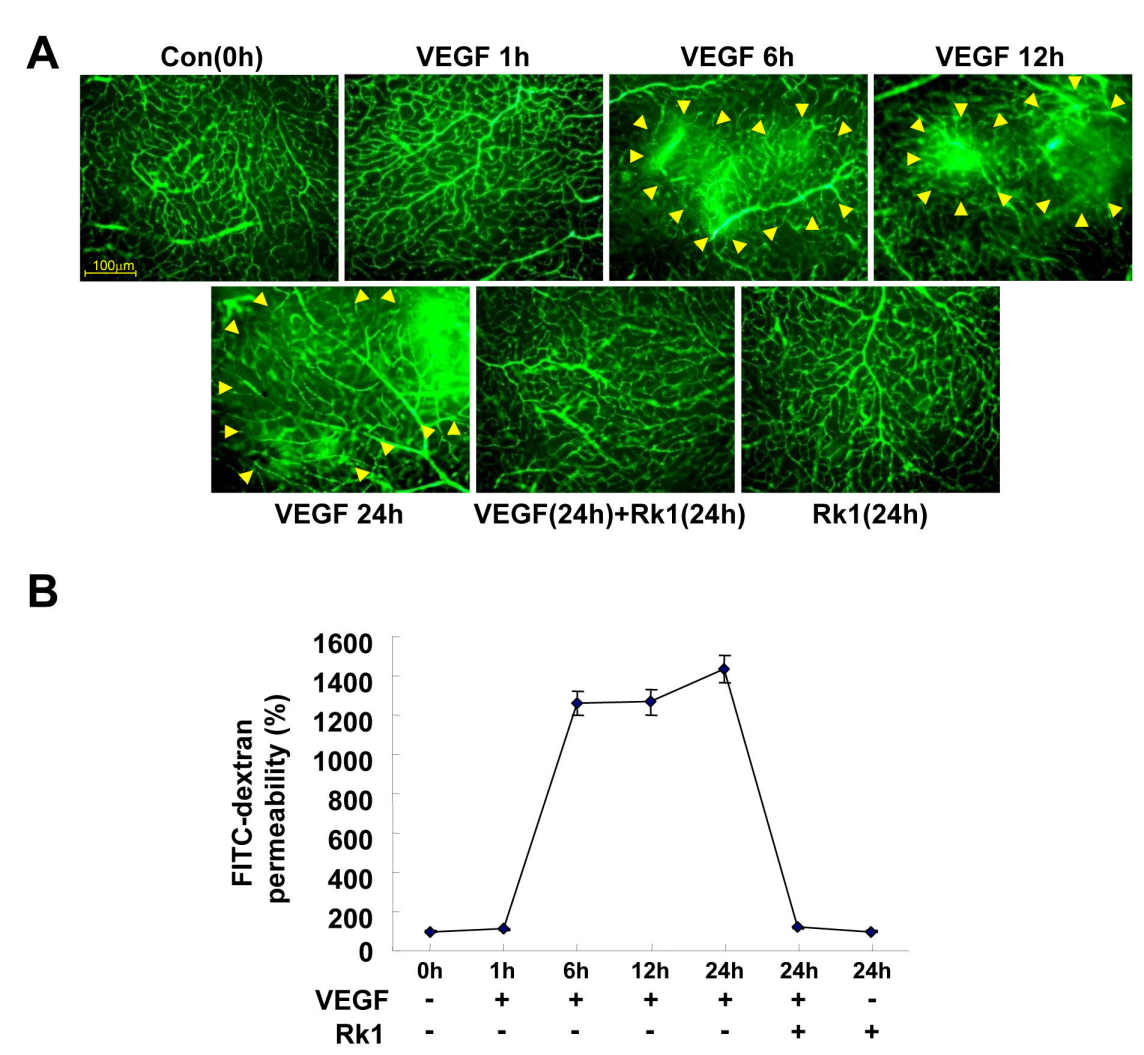
Rk1 inhibits vascular leakages of retina by VEGF. (**A**) Mice were injected with 50 ng VEGF alone or in combination with 15 µg Rk1 into the vitreous of one eye and with vehicle in the contra lateral eye. At indicated times after injection, 150µl FITC-Dextran (30 mg/ml in sterile PBS) was injected into the left ventricle. The retinas were then viewed using fluorescence microscopy. Yellow arrowheads indicate region of vascular permeability. All data are presented as the mean ± S.E. from three different experiments performed in duplicate. (**B**) Quantification of FITC-dextran leakage by a multi-gauge software (Fuji). Data are the mean ± S.E.

Increases of capillary permeability are mainly attributed to the breakdown of the BRB. The major component of the BRB is the TJ complex, which plays an important role in controlling the paracellular flux of molecules. To address whether or not the inhibitory effect of Rk1 on VEGF-induced vasopermeability is due to changes in the levels of TJ protein expression, we examined the transcription levels of a subset of representative TJ proteins (ZO-1, ZO-2, and occludin). Unfortunately, the mRNA levels of these proteins were not altered by Rk1 treatment (Figure 2A), ruling out the possibility that Rk1 directly regulates the transcription of TJ proteins. It has been reported that the TJ protein complex is stabilized by close connection to the actin cytoskeleton in the membrane region and VEGF is suggested to destabilize this complex through endocytosis [3]. Therefore, we decided to test the effect of Rk1 on the stability of TJ proteins. In untreated confluent HRECs, TJ proteins were predominantly present in the membrane fraction. Interestingly, we found that VEGF treatment increased the proportion of TJ proteins in the Triton-X-100 soluble cytosolic fraction with a reciprocal decrease in the amount of TJ proteins in the membrane fraction. Furthermore, we discovered that this intracellular re-localization was blocked by Rk1 pretreatment (Figure 2B, 2C). In order to determine the significance of this important finding, we examined the effect of Rk1 on TJ protein by using immunostaining techniques. Normally, confluent HRECs display a polygonal shape and linear pattern of TJ proteins at the cell borders and this characteristic localization of TJ proteins is disrupted by VEGF treatment (Figure 3A, 3B). Interestingly, pretreatment with Rk1 effectively inhibits this VEGF effect on TJ protein localization (Figure 3A, 3B). Given our previous observation, we wondered if Rk1 could inhibit VEGF-induced vascular leakage *in vivo*. To this end, we co-injected VEGF and Rk1 into the vitreous cavities of mice and performed fluorescein angiography 24 h post injection. To our surprise, we found that in deed Rk1 significantly inhibits VEGF-induced fluorescein leakage in the mouse retina (Figure 4A, 4B). Collectively, these results demonstrate that Rk1 has a strong capacity to prevent VEGF-induced vascular hyper-permeability and retinal vascular leakage by stabilizing endothelial TJ proteins at cell to cell contacts.

### Rk1 prevents AGE-induced retinal endothelial permeability

**Figure 5:**
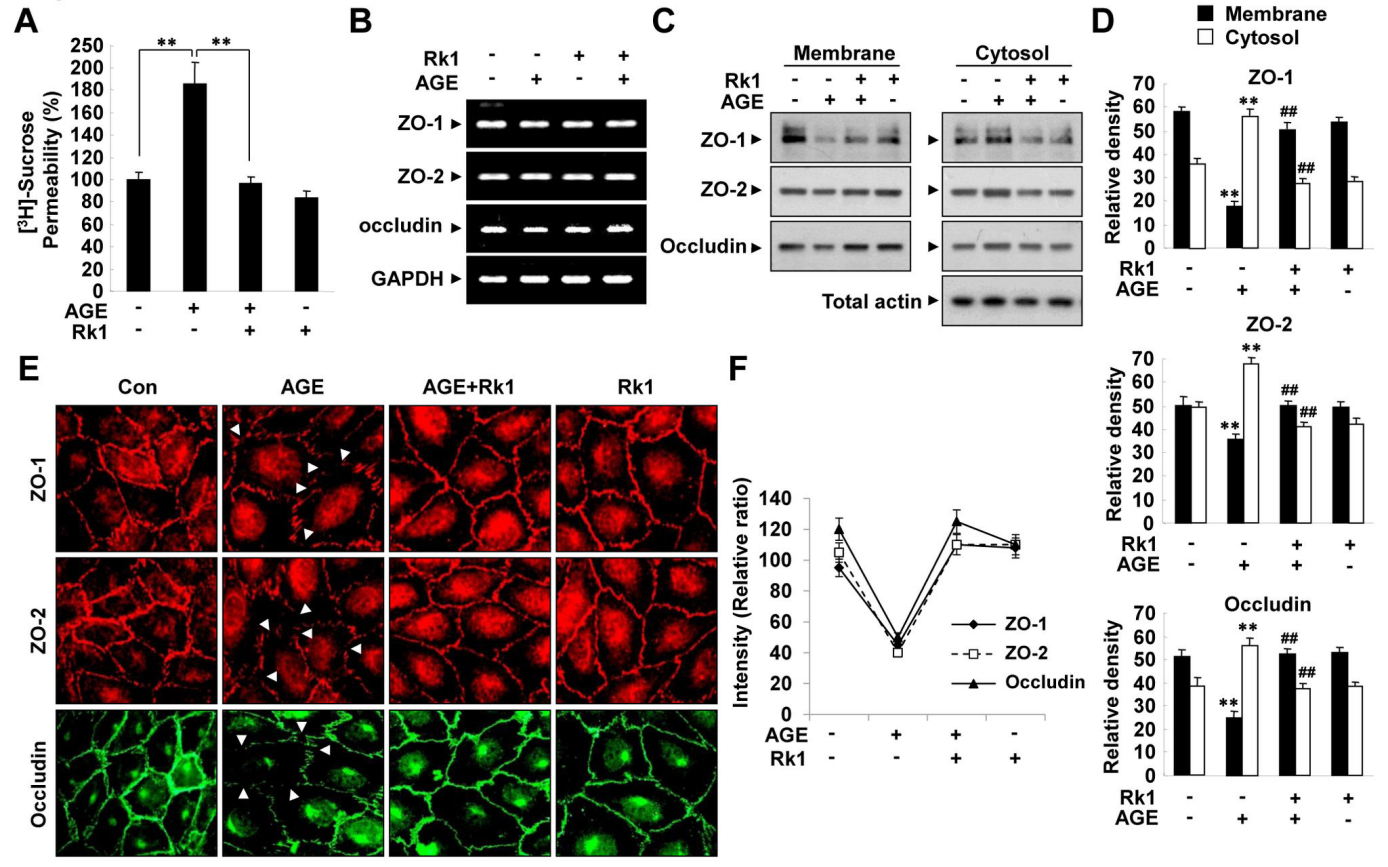
Rk1 inhibits AGE-induced retinal endothelial permeability. (**A**) HRECs were plated onto a Transwell filter . After reaching confluence, HRECs were pretreated for 40 min with or without Rk1 (10 µg/ml) prior to stimulation with 5 µM AGE-BSA for 10 h. HRECs were cultured with 50 µl (0.8 µCi/ml) of [^3^H] sucrose (1 µCi/µl) added to the upper compartment. The amount of radioactivity that diffused into the lower compartment was determined after 30 min by liquid scintillation counter. Data are the mean ± S.E. **, *P* < 0.01 *vs*. AGE alone. (**B**) HRECs were pretreated for 40 min with or without Rk1 (10 µg/ml) prior to stimulation with 5 µM AGE-BSA for 10 h. Total mRNAs were isolated and RT-PCR was performed with specific primers for human ZO-1, ZO-2, and occludin. GAPDH served as an internal control. (**C**) Confluent HRECs were pretreated for 40 min with or without Rk1 (10 µg/ml) prior to stimulation with 5 µM AGE-BSA for 10 h. Western blots were probed with anti-ZO-1, anti-ZO-2, anti-occludin and anti-Actin. (**D**) Densitometric analyses are presented as the relative ratio of tight junction proteins in membrane and cytosol and quantified with NIH ImageJ software. (**E**) Confluent HRECs were pretreated for 40 min with or without Rk1 (10 µg/ml) prior to stimulation with 5 µM AGE-BSA for 10 h. The cells were then fixed and stained with anti-ZO-1, anti-ZO-2, and anti-occludin antibodies. Arrowheads indicate disruption of tight junction proteins. (**F**) Tight junction protein intensity at cell borders was analyzed and quantified with NIH ImageJ software. Data are the mean ± S.E. **, *p* < 0.01 *vs*. Control. ^##^, *p* < 0.01 *vs*. AGE alone.

**Figure 6:**
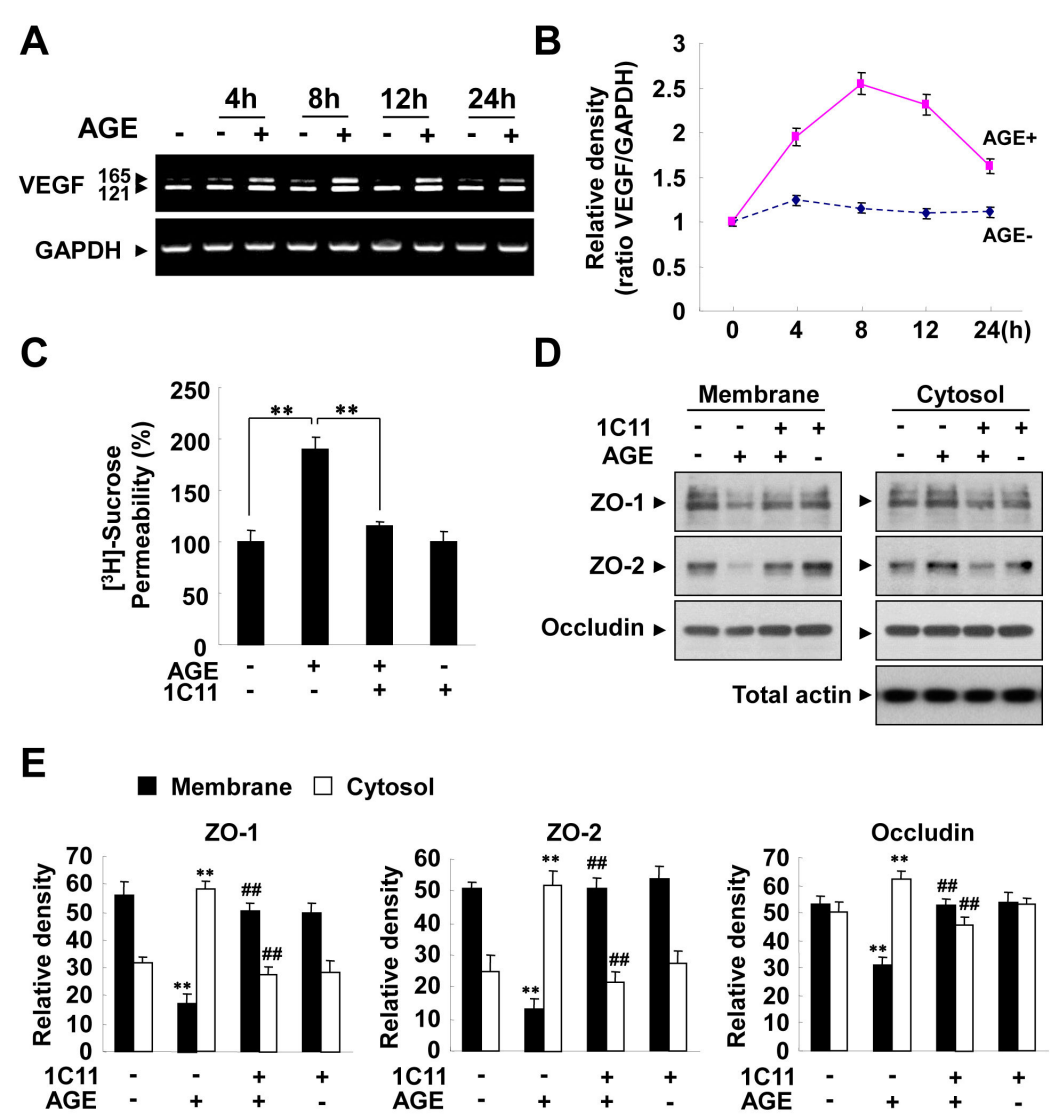
AGE induces retinal endothelial permeability *via* expression of VEGF. (**A**, **B**) HRECs were stimulated with 5 µM AGE-BSA for the indicated times. Total mRNAs were isolated and RT-PCR was performed with specific primers for human VEGF. GAPDH served as an internal control. (B) Ratio of VEGF mRNA to GAPDH mRNA. (**C**) HRECs were plated onto a Transwell filter. After reaching confluence, HRECs were pretreated for 30 min with or without 1C11 (10 µg/ml) prior to stimulation with 5 µM AGE-BSA for 10 h. HRECs were cultured with 50 µl (0.8 µCi/ml) of [^3^H] sucrose (1 µCi/µl) added to the upper compartment. The amount of radioactivity that diffused into the lower compartment was determined after 30 min with a liquid scintillation counter. Data are the mean ± S.E. **, *p* < 0.01 *vs*. AGE alone. (**D**) Confluent HRECs were incubated for 30 min with or without 10 µg/ml 1C11 and stimulated with 5 µM AGE-BSA for 10 h. Translocations of TJ proteins were assessed, as described in “Experimental Procedures”. The Triton X-100-insoluble and soluble fractions were subjected to SDS-polyacrylamide gel electrophoresis followed by Western blot analysis with anti-ZO-1, anti-ZO-2, and anti-occludin antibodies. Blots are representative of three independent experiments. (**E**) Densitometric analyses are presented as the relative ratio of tight junction proteins in membrane and cytosol and quantified with NIH ImageJ software. Data are the mean ± S.E. **, *p* < 0.01 *vs*. control. ^##^, *p* < 0.01 *vs*. AGE alone.

The formation of Advanced Glycation End-product (AGE) promoted by hyperglycemia has been suggested to be important in the development of DR by increasing vessel leakiness [24,25]. Therefore, we sought to determine whether Rk1 had an effect on AGE-induced vascular hyper-permeability. AGE increased the permeability of HRECs *in vitro*, an effect that was blocked by Rk1 treatment (Figure 5A). Consistently, AGE significantly decreased the protein levels and membrane localization of TJ proteins and these effects were also blocked by pretreatment of Rk1 (Figures 5C, 5D, 5E, 5F). However, AGE had no significant effect on the mRNA levels of TJ proteins (Figure 5B). Several lines of evidence suggest that AGE contributes to the pathogenesis of DR by inducing VEGF expression in the retina. Treatment of HRECs with AGE increased the VEGF mRNA level within 4 h and this level was sustained for up to 24 h (Figure 6A, 6B). To determine whether AGE-induced vascular hyper-permeability is due to the expression of VEGF, we treated cells with VEGF receptor 2 (VEGFR2; KDR) neutralizing antibody (1C11) before AGE treatment. VEGFR2-neutralizing antibody inhibited the AGE-induced HREC permeability and re-distribution of TJ proteins from the membrane fraction to the cytosolic fraction (Figure 6C, 6D, 6E), suggesting that VEGF expression at least partially contributes to the AGE-induced vascular hyper-permeability.

**Figure 7:**
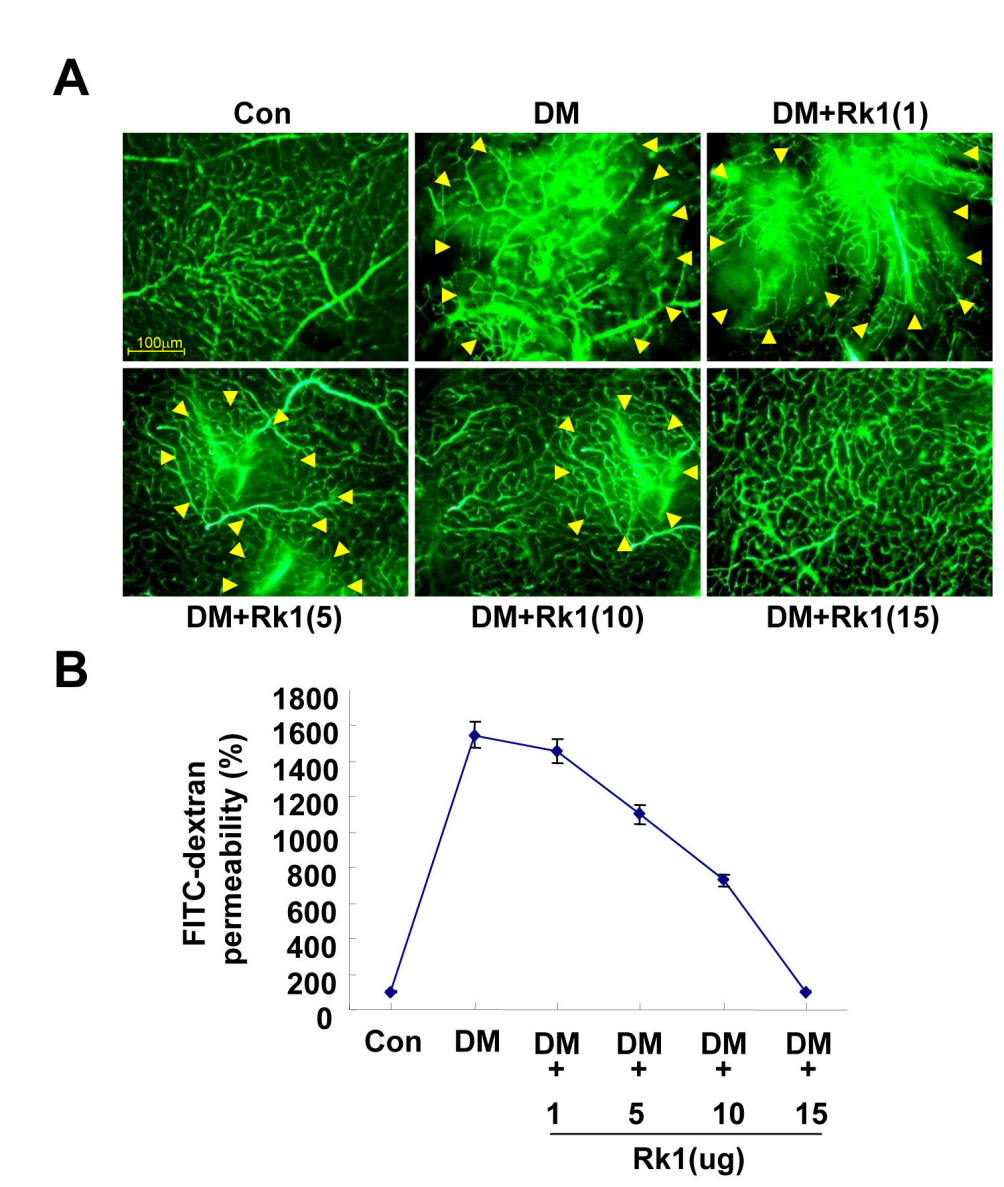
The effect of Rk1 on retinal vascular leakage of a DM model. (**A**) Diabetic mice were injected with Rk1 (1, 5, 10, 15µg) into the vitreous of one eye and with vehicle in the contra lateral eye. At 24 h after injection, 150 µl FITC-Dextran (30 mg/ml in sterile PBS) was injected into the left ventricle. The retinas were then viewed by fluorescence microscopy. Yellow arrowheads indicate region of vascular permeability. (**B**) Quantification of FITC-dextran leakage by a multi-gauge software (Fuji). Data are the mean ± S.E.

**Figure 8:**
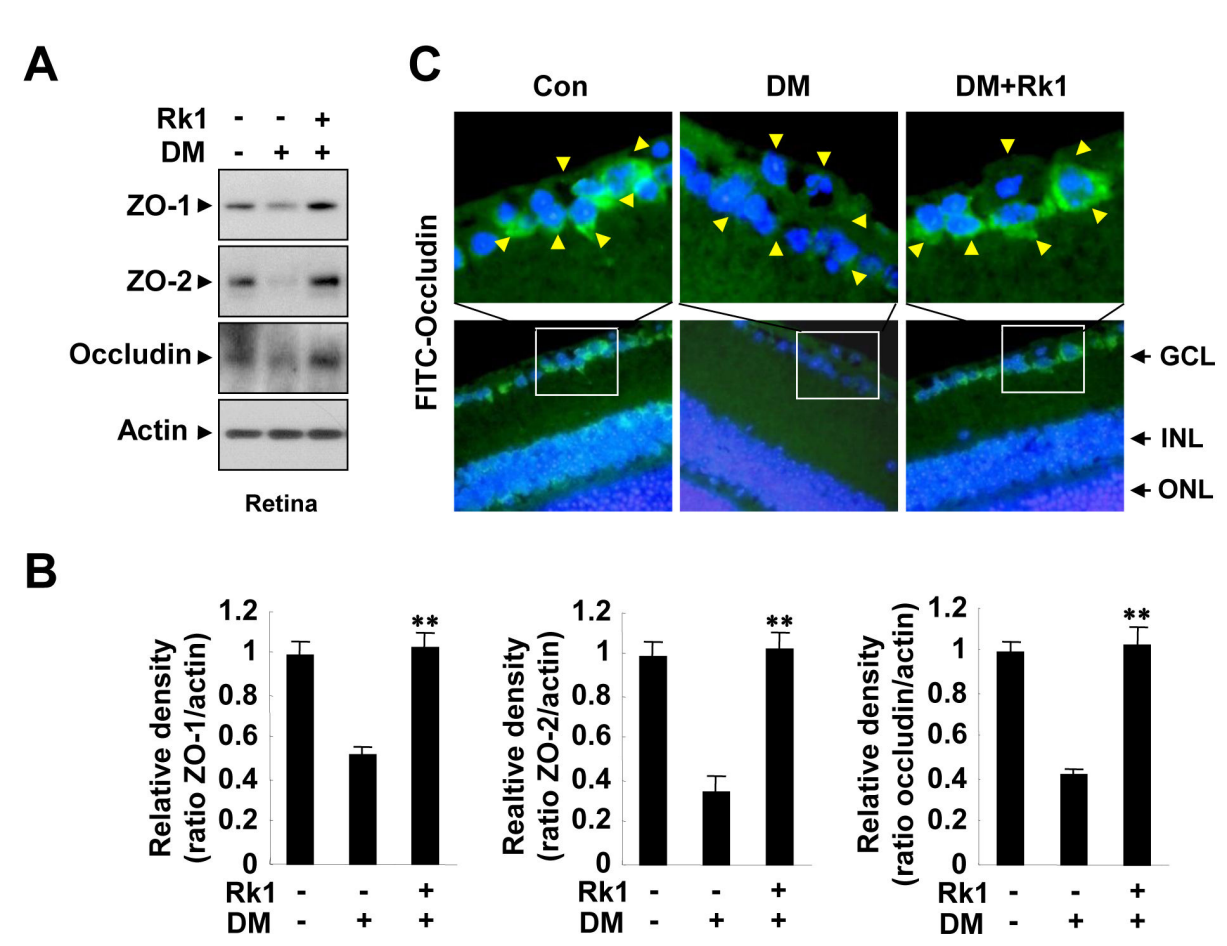
Rk1 inhibits occludin reduction of a DM model. (**A**) Diabetic mice were injected with 15 µg Rk1 into the vitreous of one eye and with vehicle in the contra lateral eye. At 24 h after injection, eye homogenate was subjected to SDS-polyacrylamide gel electrophoresis, and immunoblotting with mouse anti-ZO-1, anti-ZO-2, and anti-occludin antibodies. (**B**) Densitometric analyses are presented as the relative ratio of tight junction proteins in membrane and cytosol and quantified with NIH ImageJ software. (**C**) Paraffin sections (4 µm thick) of retina were stained with anti-occludin antibodies. Nuclei were counterstained with DAPI and are seen in blue. Yellow arrowheads indicate retina blood vessel. GCL, ganglion cell layer; INL, inner nuclear layer; ONL, outer nuclear layer. Data are the mean ± S.E. **, *P* < 0.01 *vs*. DM alone.

To further investigate whether Rk1 inhibits vascular leakage of DR *in vivo*, we created a diabetic mouse (DM) model, in which hyperglycemia is induced with streptozotocin and measured vascular leakage using fluorescein angiography. Streptozotocin-induced hyperglycemia caused fluorescein leakage from retina vessels in the mice, while fluorescein was restricted to vascular lumen in control mice (Figure 7A, 7B). Intravitreal injection of Rk1 blocked the vascular leakage in a DM model (Figure 7A, 7B). Moreover, streptozotocin-induced hyperglycemia significantly decreased the level of TJ proteins in retina, which was inhibited by Rk1 treatment (Figure 8A, 8B). In addition, mouse retinal immunofluorescent staining revealed a significant reduction of occludin level in DM mice compared to control mice. The injection of Rk1 into the vitreous cavities of DM mice was able to induce recovery levels of occludin in retina comparable to TJ protein levels of control mice (Figure 8C). Taken together, these results suggest that Rk1 treatment may be able to control BRB breakdown and vascular leakage in DR.

### Rk1 blocks stress fiber formation induced by VEGF or AGE in HRECs

**Figure 9:**
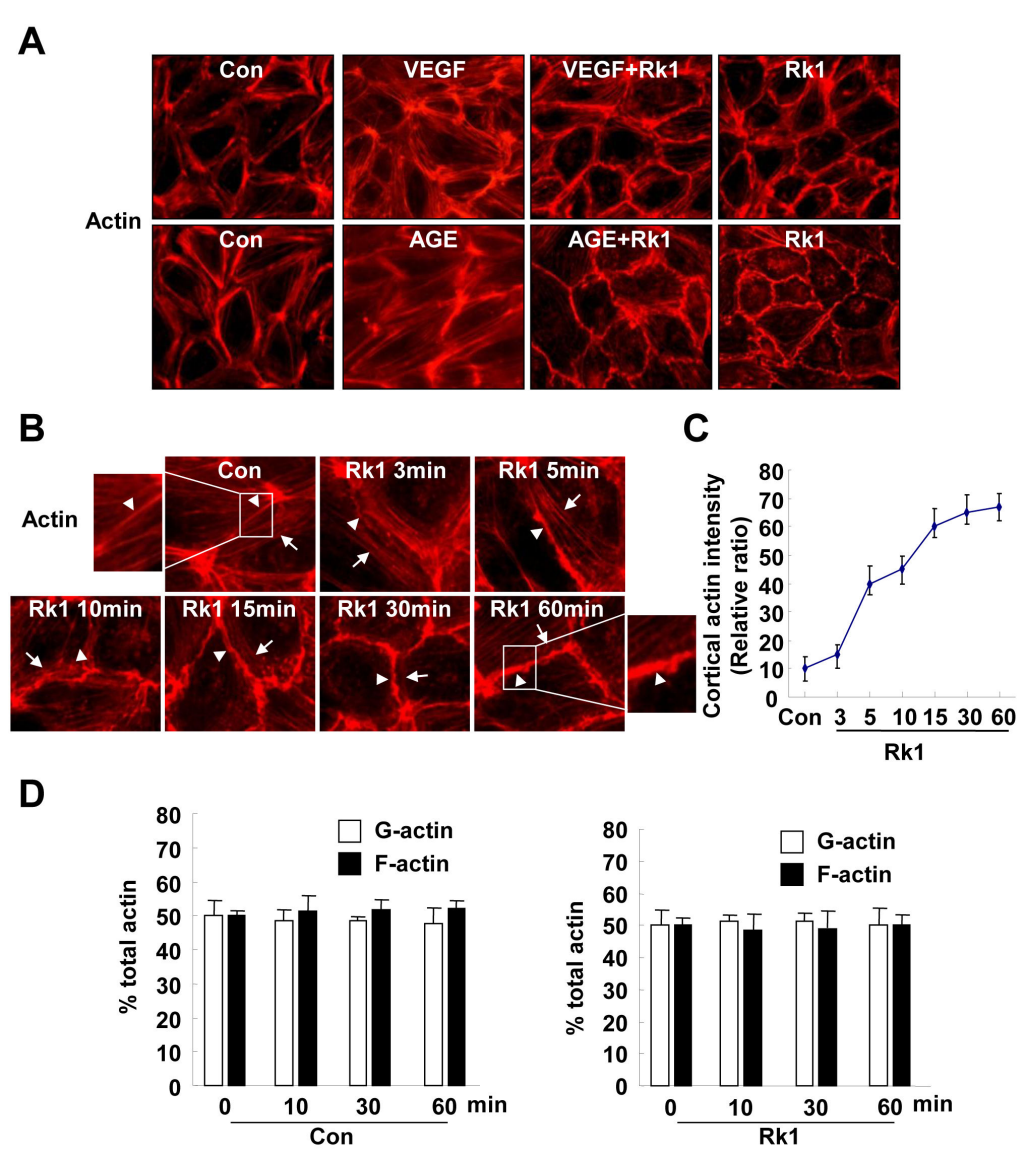
Rk1 promotes cortical actin ring formation in HRECs. (**A**) Confluent HRECs were pretreated for 40 min with or without Rk1 (10 µg/ml) prior to stimulation with 20 ng/ml VEGF (1 h) or 5 µM AGE-BSA (10 h). (**B**) Confluent HRECs were treated with Rk1 (10 µg/ml) for the indicated times. The cells were then fixed and processed for immunofluorescence staining using Rhodamine Phalloidin. Arrowheads show junctional actin, arrows point to thin bundles. (**C**) Cortical actin intensity was analyzed and quantified with NIH ImageJ software. (**D**) Global levels of G-actin and F-actin pools were quantified by immunofluorescence in a time course after treatment of Rk1 (10 µg/ml). HRECs were stained with DNAse1 (G-actin) and phalloidin (F-actin) and the whole optical field quantified for each fluorophore. Values are expressed as percentage of total actin. Data are the mean ± S.E.

Endothelial barrier regulation critically depends on the dynamics of the endothelial cell actin cytoskeleton organization [3,5–7]. To further understand the molecular mechanism of Rk1 in regulating endothelial cell junctions, we examined the spatial localization of actin filaments by immunofluorescence. Control HRECs showed marginally positioned ring-like bundles of F-actin filaments and a few stress fibers aligned with the major axis of the cell (Figure 9A). VEGF or AGE treatment decreased the amount of cortical actin ring structures and increased the actin stress fibers in the cells (Figure 9A). The re-organization of the actin cytoskeleton in VEGF- or AGE-treated HRECs was inhibited by Rk1 pretreatment (Figure 9A). Notably, Rk1 alone was sufficient to induce the formation of cortical actin ring structures at cell borders in a time-dependent manner (Figure 9B, 9C). However, the increased assembly of actin into the cortical ring structure at regions of cell-cell contact after Rk1 treatment was not due to an overall enhancement of actin polymerization, since the ratio of monomeric and filamentous actin did not significantly vary after Rk1 stimulation (Figure 9D). These results suggest that Rk1 induces the spatial re-organization of the actin cytoskeleton rather than promoting actin polymerization to increase cortical actin ring structure.

**Figure 10:**
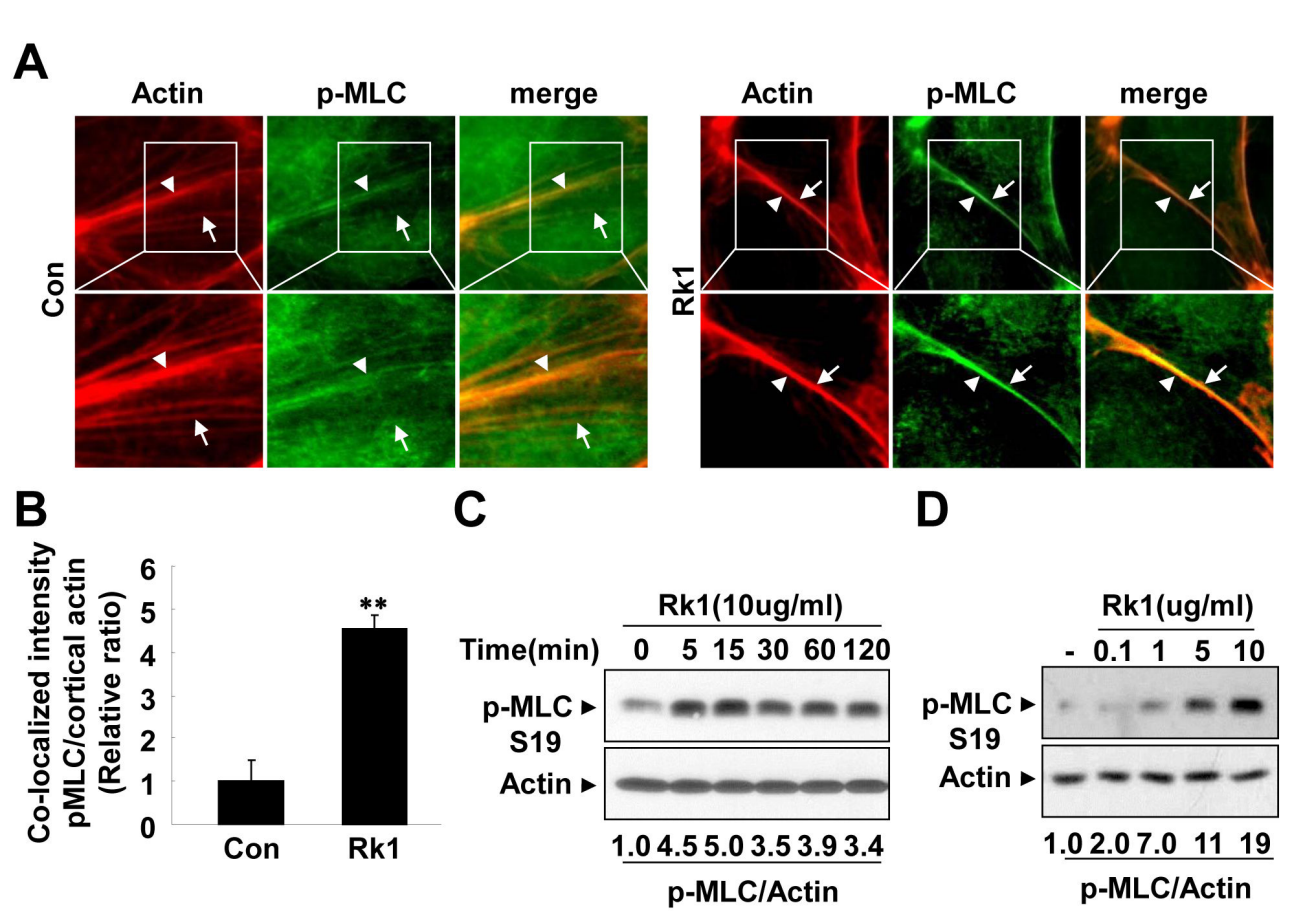
Rk1 induces phosphorylation of MLC and co-localization of pMLC with the cortical actin ring. (**A**) Confluent HRECs were treated with Rk1 (10 µg/ml) for 1h. The cells were then fixed and processed for immunofluorescence staining using Rhodamine Phalloidin and anti-pMLC (S19) antibodies. The samples were viewed by fluorescence microscopy. Arrowheads show junctional actin, arrows point to thin bundles and localization of pMLC. (**B**) Co-localization intensity of pMLC and junctional actin was analyzed and quantified with NIH ImageJ software. (**C**, **D**) Confluent HRECs were treated with Rk1 (10 µg/ml) for indicated times and stimulated with Rk1 (0.1, 1, 5, or 10 µg/ml) for 15 min. Western blot was performed using an antibody specific for phospho-MLC (S19). Blot was reprobed with an anti-actin antibody to verify equal loading of protein in each. Densitometric analyses are presented as the relative ratio of p-MLC and Actin and quantified with NIH ImageJ software. Data are the mean ± S.E. **, *p* < 0.01 *vs*. Control.

Since myosin function, which is regulated by phosphorylation, is involved in the stability of the cortical actin ring structure [12], we decide to examine the phosphorylation status of the MLC. Interestingly, we found that control cells had minimal amounts of p-MLC localized to peripheral actin bundles. Rk1 treatment dramatically increased the amount of p-MLC co-localized with the cortical actin ring structures (Figure 10A, 10B). Furthermore, Rk1 treatment led to phosphorylation of MLC and maximal activation was observed after 15 min (Figure 10C). Rk1 also increased the phosphorylation of MLC in a concentration-dependent manner (Figure 10D). Taken together, these results suggest that Rk1 may enhance endothelial barrier integrity by inducing the reorganization of the actin cytoskeleton into a cortical ring structure with co-localized p-MLC.

### Rk1 induces cortactin phosphorylation

**Figure 11:**
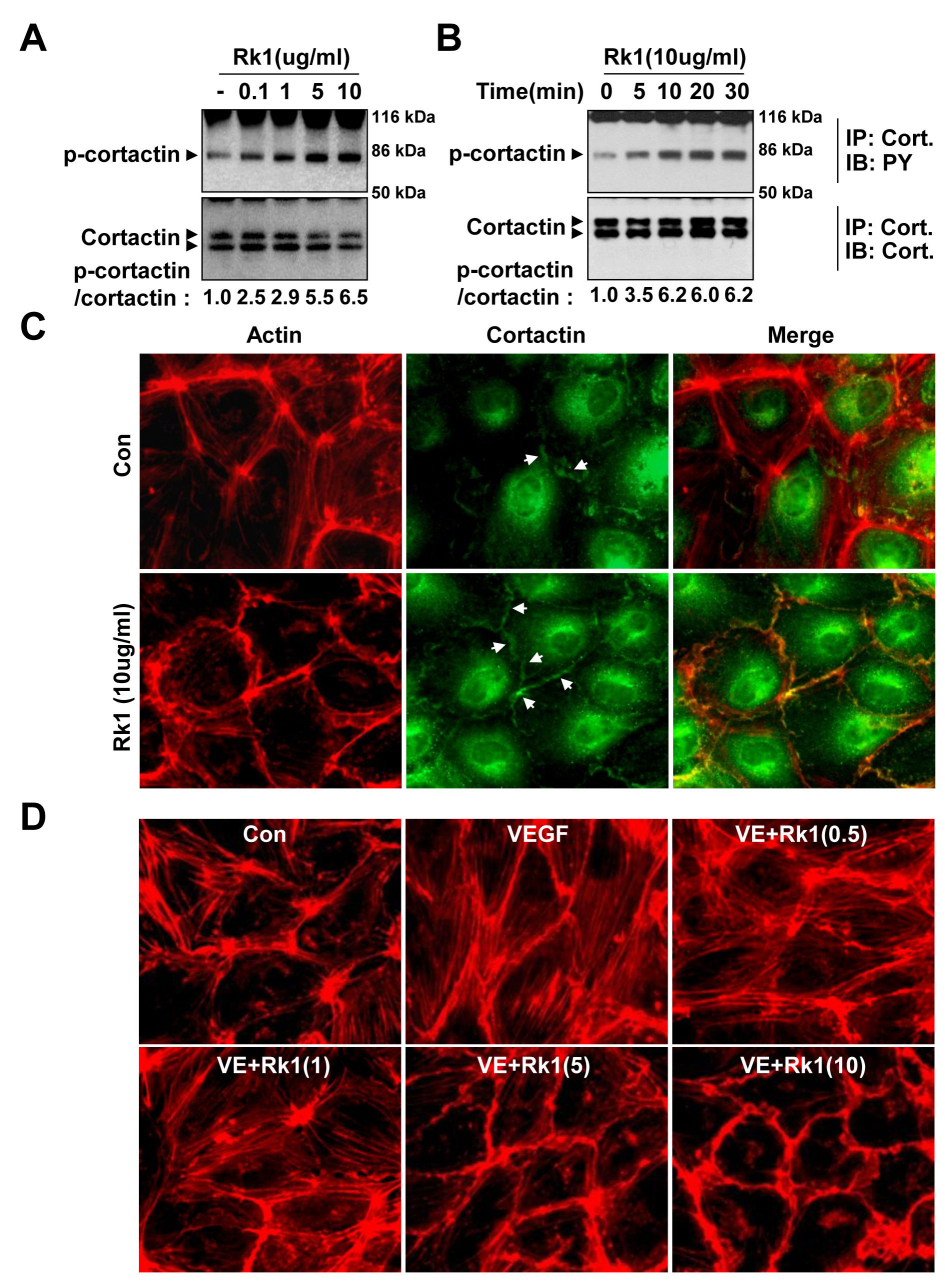
Rk1 induces cortactin phosphorylation. (**A**, **B**) Confluent HRECs were treated with Rk1 (0.1, 1, 5, or 10 µg/ml) for the indicated times. Cell lysates were subject to affinity precipitation using anti-cortactin antibody. Total cortactin indicates total amount of active and inactive cortactin in the HRECs. Western blot was performed using an antibody specific for phospho-tyrosine (PY) and cortactin (Cort). (**C**) Confluent HRECs were treated with Rk1 (10 µg/ml) for 30 min. The cells were then fixed and stained with Rhodamine Phalloidin and anti-cortactin antibody. Arrows indicate cortactin. (**D**) Confluent HRECs were pretreated for 1 h with or without VEGF (20 ng/ml) prior to stimulation with Rk1 (0.5, 1, 5, or 10 µg/ml) for 1 h. The cells were then fixed and processed for immunofluorescence staining using Rhodamine Phalloidin. The samples were viewed by fluorescence microscopy.

Cortactin, an actin filament-binding protein, has emerged as a central element connecting signaling pathways with cytoskeleton restructuring particularly during cortical ring formation [14]. We examined the effect of Rk1 on the phosphorylation of cortactin. Rk1 treatment significantly increased the tyrosine phosphorylation of cortactin in a concentration- and time-dependent manner (Figure 11A, 11B). Furthermore, immunostaining against cortactin showed that Rk1 induced the translocation of cortactin to the cellular periphery, co-localizing with the cortical actin (Figure 11C). Rac small GTPase is important in the regulation of the cortical actin structure [14,26]. GTP bound Rac interacts with WAVE, a WASP related polypeptide, resulting in the activation of Arp2/3 and the subsequent recruitment of cortactin to the cell cortical area [27]. We consistently found that stimulation of HRECs with Rk1 activated Rac, with the peak activation occurring within 5 minutes (Figure S1). Taken together, these results suggest that cortactin translocation may be involved in the formation of the Rk1-induced cortical actin ring formation. In order to be an efficient therapeutic agent for the treatment of disease states, Rk1 must be able to restore damaged endothelial cell junctions. To investigate whether Rk1 had the ability to reverse VEGF-induced actin stress fiber formation, we added Rk1 after treatment of VEGF to HRECs. As shown in Figure 11D, Rk1 treatment shifted VEGF-induced actin stress fibers into cortical actin ring structures in a dose-dependent manner. These results suggest that Rk1 may reverse VEGF-induced vascular leakage by promoting the formation of cortical actin ring

### Rk1 inhibits thrombin or histamine-induced retinal endothelial permeability

**Figure 12:**
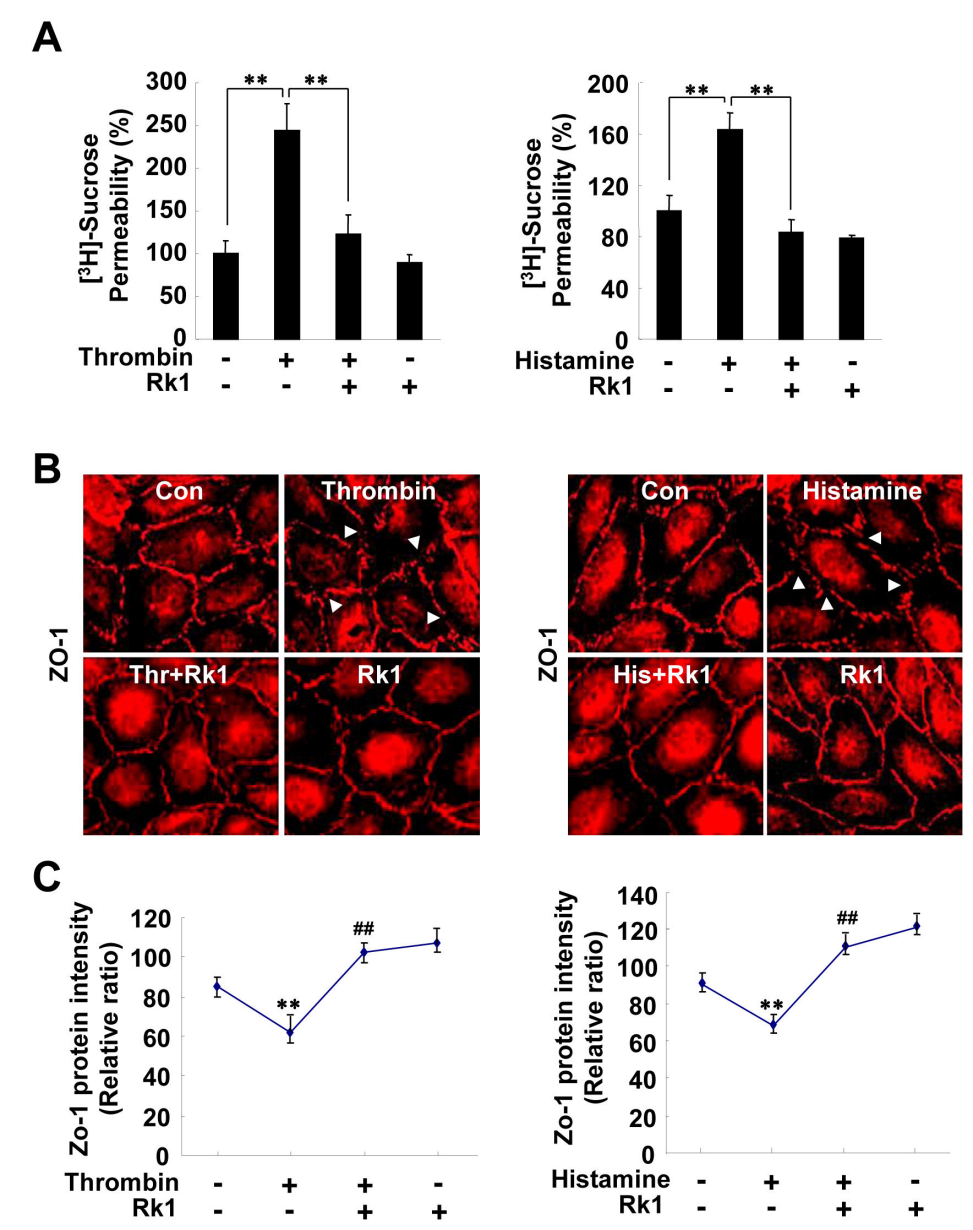
Rk1 inhibits thrombin or histamine-induced retinal endothelial permeability. (**A**) HRECs were plated onto a Transwell filter . After reaching confluence, HRECs were pretreated for 40 min with or without Rk1 (10 µg/ml) prior to stimulation with Thrombin (10 units/ml) or Histamine (100 µM) for 1 h. [^3^H] sucrose permeability assay was performed. Data are the mean ± S.E. **, *P* < 0.01 *vs*. Thrombin or Histamine alone. (***B***) Confluent HRECs were pretreated for 40 min with or without Rk1 (10 µg/ml) prior to stimulation with Thrombin (10 units/ml) or Histamine (100 µM) for 1 h. The cells were then fixed and stained with anti-ZO-1 antibody. Arrowheads indicate disruption of ZO-1 proteins. (**C**) ZO-1 protein intensity at cell borders was analyzed and quantified with NIH ImageJ software. Data are the mean ± S.E. **, *P* < 0.01 *vs*. Control. ^##^, *p* < 0.01 *vs*. Thrombin or Histamine alone.

The vasoactive mediators thrombin and histamine have long been known to increase vascular permeability under inflammatory conditions *in vitro* and *in vivo* [28,29]. Endothelial permeability induced by thrombin and histamine is accompanied by alterations in cell-cell junctions and the actin cytoskeleton [30]. We tested whether Rk1 is also capable of blocking thrombin or histamine-induced permeability and TJ protein disorganization in HRECs. Thrombin and histamine significantly increased the permeability of an HREC monolayer, an effect that was blocked by pre-treatment with Rk1 (Figure 12A). In addition, immunostaining of the TJ protein ZO-1 revealed that Rk1 inhibits thrombin or histamine-induced segmentation and disorganization of TJ proteins at cell borders (Figure 12B, 12C). Taken together, these results suggest that Rk1 may have an inhibitory effect against hyper permeability triggered by various types of stimuli.

## Discussion

More than 30 kinds of ginsenosides, the putatively active components in ginsengs, have been identified thus far and many individual ginsenosides have been studied with respect to their specific pharmacological effects in various disease models [31]. However, the structural characteristics and the precise mechanisms by which ginsenosides deliver their medicinal benefits have not been fully elucidated. This study represents important work to demonstrate that a sub-fraction of ginsenoside derivatives extracted from sun ginseng, specifically Rk1, have a surprising effect on endothelial cell barrier function and restoration.

Recent studies have shown that certain ginsenosides, depending on their structures, exert dual functions in neovascularization. Ginsenoside Rg1 promotes neovascularization *in vivo* and endothelial cell proliferation, chemo-invasion, and tubulogenesis *in vitro*, while Rb1, Rb2, and Rg3 inhibit angiogenesis [32–35]. We have also demonstrated that 20(S)-Ginsenoside Rg3 prevents endothelial cell apoptosis *via* inhibition of a mitochondrial caspase pathway [36]. Interestingly, Leung et al. have recently reported that Rb1 regulates capillary morphogenesis through binding and activation of the oestrogen β receptor in ECs [35,37]. These results suggest that individual ginsenosides have different functional roles in the vasculature and have specific agonistic or antagonistic actions depending on EC receptor context and on the side chain moieties attached to their unique sterol backbone. Our data show that Rk1, one of the main ingredients of sun ginseng, enhances the endothelial barrier integrity in HRECs, whereas both Rb1 and Rg1, which are rich in white ginseng, have no significant effect on endothelial permeability. Structural comparison of these ginsenosides suggests that the precise structural characteristics of ginsenosides, such as side chains and glycosylation at the R1 site, are essential for their vascular functions and raise the possibility that Rk1 may interface with a specific signaling axis in ECs to regulate endothelial integrity. Endothelial permeability is regulated by the balance between intracellular tension imposed by actin stress fibers and adhesive cell-cell forces imposed by intercellular junctions, which are stabilized by the cortical actin ring structure [38,39]. In addition, previous studies have shown that VEGF, thrombin, and histamine, molecules known to increase vascular permeability, markedly increase actin stress fiber formation [4,40], an observation that is consistent with our data. By comparison, endothelial barrier protective factors, such as SIP and HGF, have been shown to enhance cortical actin ring formation [11,41]. Notably, our results show that Rk1 blocked VEGF-induced stress fiber formation with a concomitant increase of cortical ring formation, resulting in reduction of permeability in ECs. Furthermore, Rk1 treatment was found to shift the actin cytoskeleton organization from stress fibers, which were pre-formed in response to VEGF stimulation, towards cortical ring formation, indicating that Rk1 is capable of re-organizing the actin cytoskeleton. Rk1 treatment also enhances stability and positioning of TJ proteins at cell-cell contacts, coincident with cortical actin ring formation. These findings indicate that the EC barrier protective effect of Rk1 is likely due to its ability to induce cortical actin ring formation and subsequent stabilization of TJ complexes at cell contact regions.

Our data also illustrate the signaling mechanism by which Rk1 induces and stabilizes the cortical actin ring structure in ECs. Depending on where it is activated, MLCK appears to participate in the enhancement or disruption of endothelial barrier integrity [11]. MLC phosphorylation at the cell periphery correlates well with cortical actin ring formation in ECs stimulated with EC barrier enhancers, such as S1P [12]. Furthermore, cortactin interacts with MLCK *via* its SH3 domain, and this interaction is important for MLC phosphorylation and EC barrier regulation [13]. However, MLC phosphorylation is also required for the stress fiber formation associated with the disruption of endothelial integrity induced by VEGF and other endothelial permeability factors [40,42]. Therefore, coordinate spatial regulation of MLCK and cortactin appears to be important for the specific rearrangement of the actin cytoskeleton and co-activation of these molecules at cell contacts is likely to be essential for enhanced EC barrier integrity. Notably, our data show that Rk1 increases phosphorylation and translocation of MLC as well as cortactin to the cell periphery, corresponding to the cortical actin ring, while VEGF, an inducer of stress fiber formation, only activates MLC phosphorylation (Figure S2). Furthermore, our data show that Rk1 induces the activation of Rac GTPase in ECs. Collectively, our findings demonstrate that the barrier enhancing action of Rk1 is closely correlated with the cortical actin ring formation and junctional protein stability, both of which are critical for EC barrier formation (Figure S3).

In summary, the present study demonstrates that Rk1 is a novel endothelial barrier enhancer, which is capable of preventing or even reversing VEGF-induced vasopermeability in ECs. Notably, our study shows that Rk1 blocks the EC permeability induced not only by VEGF, but also by thrombin and histamine, highlighting the effectiveness of Rk1 in protecting vessel integrity. Despite the significance of endothelial leakage in the pathogenesis of vascular diseases a very effective pharmacological treatment has yet to be developed. Thus, the novel EC barrier protective property of Rk1 characterized in the current study may be valuable for the development of pharmaceuticals that might effectively control the pathologic vascular leakage in these diseases.

## Supporting Information

Figure S1 Rk1 induces Rac activation. Confluent HRECs were treated with Rk1 (10 µg/ml) for the indicated times. Cell lysates were subject to affinity precipitation using PAK-1 (p21-activated kinase) PBD (p21-binding domain) in recombinant protein agarose, which specifically precipitates active Rac (Rac-GTP). “Total Rac” indicates total amount of active and inactive Rac in the HREC. Western blot was performed using an antibody specific for Rac.
(TIF)

Figure S2 VEGF induces phosphorylation of myosin light chain. (TIF)

Figure S3 A schematic diagram of a proposed action mechanism of Rk1 on vascular permeability. (TIF)
