# Supplementary figures and images for: Rk1, a Ginsenoside, Is a New Blocker of Vascular Leakage Acting through Actin Structure Remodeling
Source: PLoS One. 2013 Jul 22;8(7):e68659. doi: 10.1371/journal.pone.0068659 (PMC3718811; doi:10.1371/journal.pone.0068659)

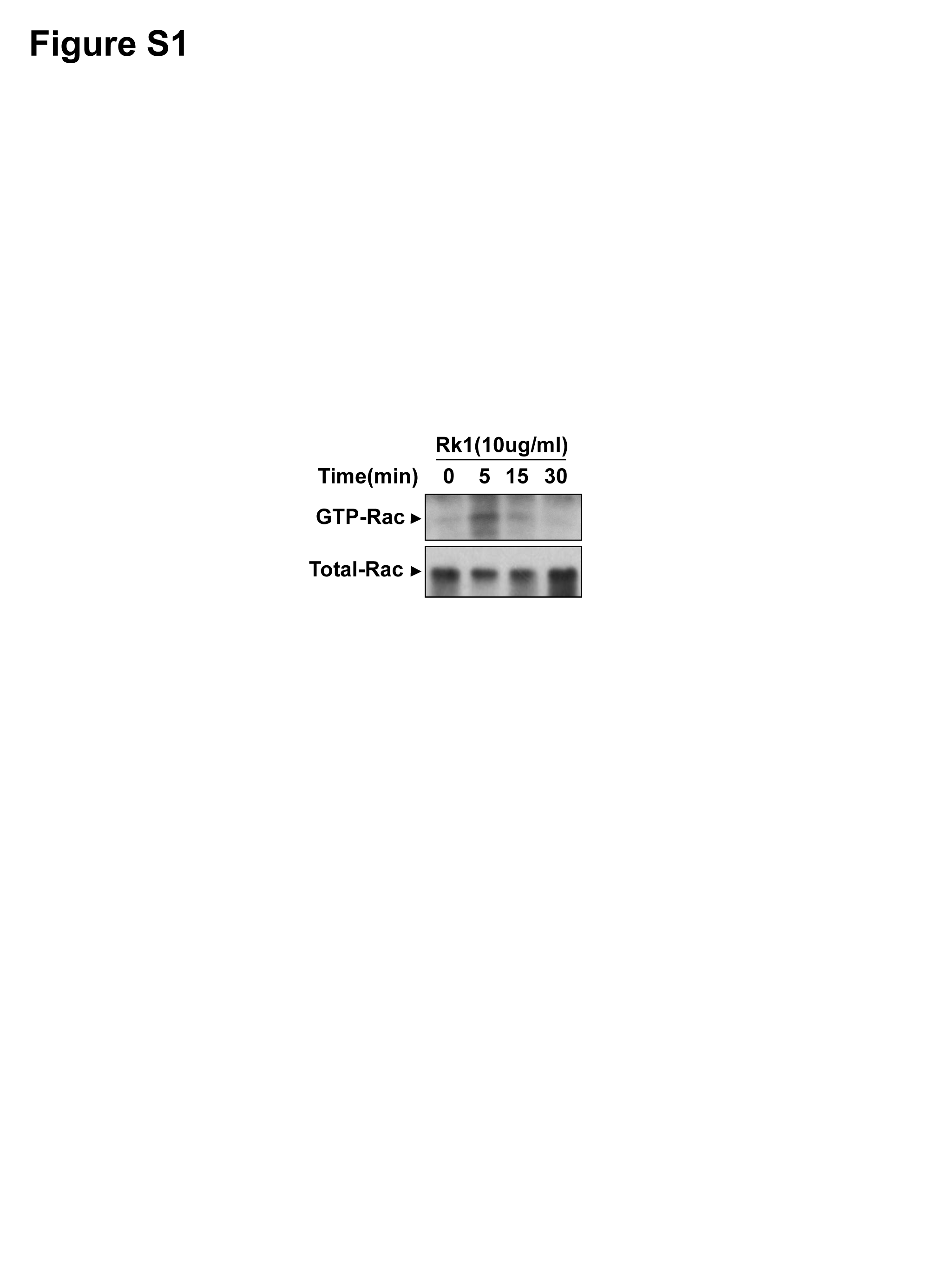

Supplement: Figure S1 — Confluent HRECs were treated with Rk1 (10 µg/ml) for the indicated times. Cell lysates were subject to affinity precipitation using PAK-1 (p21-activated kinase) PBD (p21-binding domain) in recombinant protein agarose, which specifically precipitates active Rac (Rac-GTP). “Total Rac” indicates total amount of active and inactive Rac in the HREC. Western blot was performed using an antibody specific for Rac. (TIF) [file pone.0068659.s001.tif]

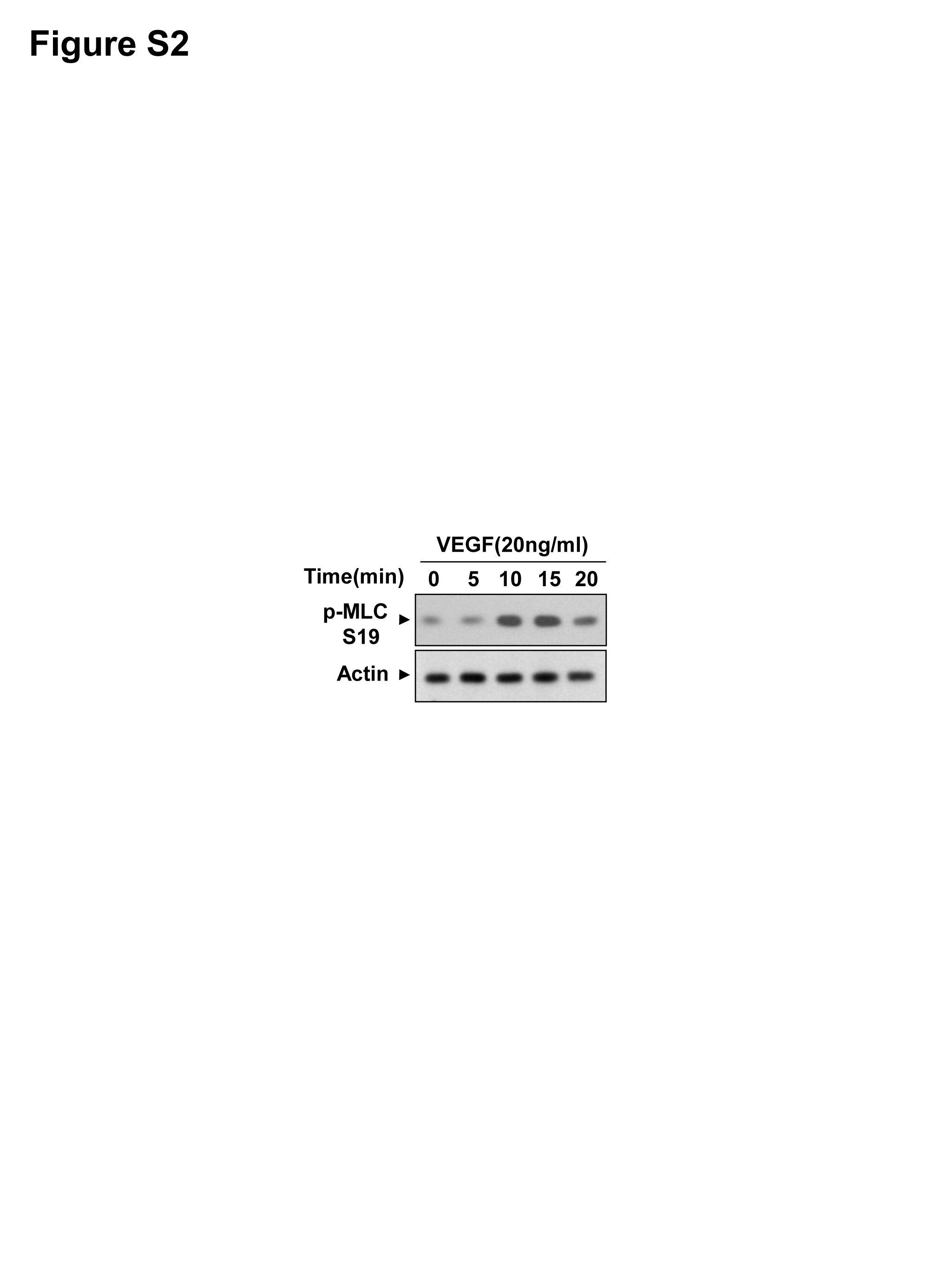

Supplement: Figure S2 — (TIF) [file pone.0068659.s002.tif]

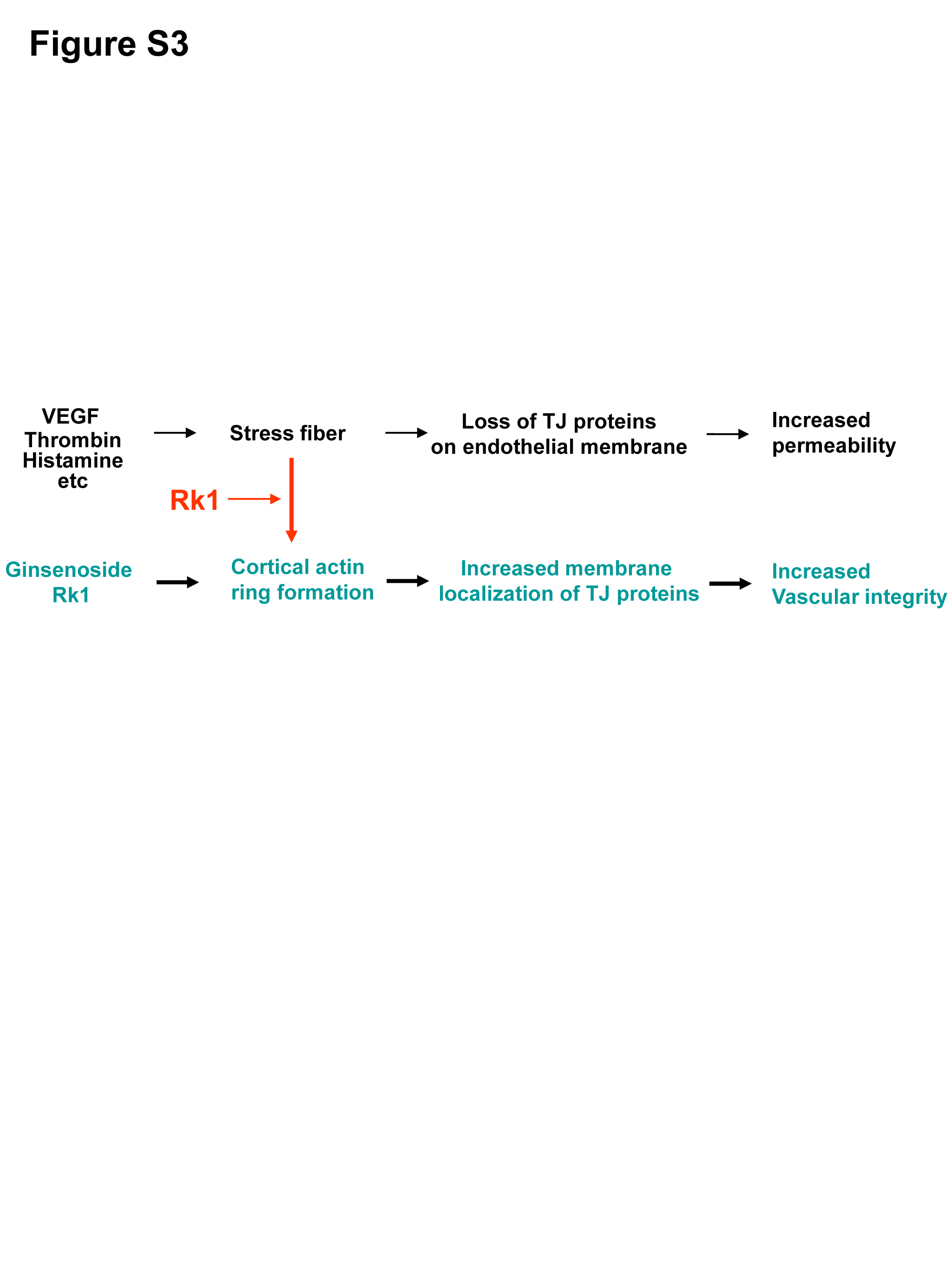

Supplement: Figure S3 — (TIF) [file pone.0068659.s003.tif]
